# DVH Analytics: A DVH database for clinicians and researchers

**DOI:** 10.1002/acm2.12401

**Published:** 2018-07-21

**Authors:** Dan Cutright, Mahesh Gopalakrishnan, Arkajyoti Roy, Aditya Panchal, Bharat B. Mittal

**Affiliations:** ^1^ Department of Radiation Oncology Warren Alpert Medical School Rhode Island Hospital Brown University Providence RI USA; ^2^ Department of Radiation Oncology Robert H. Lurie Comprehensive Cancer Center Northwestern Memorial Hospital Northwestern University Feinberg School of Medicine Chicago IL USA; ^3^ Department of Applied Statistics and Operations Research Bowling Green State University Bowling Green OH USA; ^4^ Department of Management Science and Statistics University of Texas at San Antonio San Antonio TX USA; ^5^ Department of Radiation Oncology Northwestern Medicine Chicago Proton Center Warrenville IL USA

**Keywords:** data analytics, database, DICOM, DVH, radiation therapy

## Abstract

In this study, we build a vendor‐agnostic software application capable of importing and analyzing non‐image‐based DICOM files for various radiation treatment modalities (i.e., DICOM RT Dose, RT Structure, and RT Plan files). Dose‐volume histogram (DVH) and planning data are imported into a SQL database, and methods are provided to manage, edit, view, and download data. Furthermore, the software provides various analytical tools for plan evaluations, plan comparisons, benchmarking, and plan outcome predictions. DVH Analytics is developed using Python, including libraries such as pydicom, dicompyler, psycopg2, SciPy, Statsmodels, and Bokeh for parsing DICOM files, computing DVHs, communicating with a PostgreSQL database, performing statistical analyses, and creating a web‐based user interface. This software is open‐source and compatible with Windows, Mac OS, and Linux. For proof‐of‐concept, a database with over 3,000 DVHs from a single physician's head & neck practice was built. From these data, differences in means, correlations, and temporal trends in dose to multiple organs‐at‐risk (OARs) were observed. Furthermore, an example of the predictive regression tool is reported, where a model was constructed to predict maximum dose to brainstem based on minimum distance from planning target volume (PTV) and treatment beam source‐to‐skin distance (SSD). With DVH Analytics, we have developed a free, open‐source software program to parse, organize, and analyze non‐image‐based DICOM data for use in a radiation oncology setting. Furthermore, this software can be used to generate statistical models for the purposes of quality control or outcome predictions and correlations.

## 1 INTRODUCTION

Evaluation of treatment plans in radiation oncology is heavily dependent on dose‐volume histograms (DVHs). However, such evaluations typically are limited to a small set of points from the DVH. Arguably, this is because the majority of readily available data (e.g., Emami, QUANTEC, RTOG/NRG trials, and ICRU guidelines) comprises discrete points as opposed to a continuum of data.^1^, ^2^ Comparisons with established protocols do have merit as to whether the organ‐at‐risk (OAR) criteria are met; however, from a quality control perspective, the use of historical data can better determine if a plan is atypical. Furthermore, it is conceivable that radiation toxicities may have stronger correlations to other DVH points or perhaps the combination of additional DVH points. In either case, developing a large database of treatment planning data can provide the ability of more complex statistical analysis of larger datasets more quickly and accurately than manually transcribing statistics of one plan at a time into a spreadsheet of predetermined thresholds.

To the best of our knowledge, the open‐source treatment planning and evaluation tools currently available have been developed without an explicit database. The Matlab‐based software known as CERR (Computational Environment for Radiotherapy Research) provides an open‐source platform that is effective for prototype treatment planning and evaluation, especially for research purposes. Furthermore, CERR is capable of working with patient files from DICOM or AAPM/RTOG archives, which makes data transfer between multiple platforms straightforward.^3^ For analysis across multiple patients, the Matlab‐based software DREES (dose–response explorer system) is an open‐source extension of CERR which provides a data‐driven analysis of treatment outcomes; DREES provides analytical tools such as fitting tumor control probability (TCP) and normal tissue complication probability (NTCP) curves, modeling and visualizing dose‐volume and plan metrics, and estimating uncertainty in planning parameters.^4^ The input data for DREES assume a Matlab‐based data structure comprising DVH and outcome data, which can be a limitation when working directly with DICOM files. Another option, RadOnc, is an R package designed for radiation oncology and provides an extensive library of analytical tools.^5^ However, similar to CERR and DREES, RadOnc does not provide a way to store, query, explore, or analyze a large, scalable database.

Currently, storage of patient data with open‐source platforms developed on Matlab and R is file‐based. For such individual file‐based systems, making a query on a single parameter over multiple patients can involve opening, reading, and closing a large number of files, which adds an excessive amount of memory use and computational overhead. Because of this, scalability for large datasets is a concern for currently existing open‐source platforms in radiation oncology. Ideally, a user should be able to access only the data of interest so not to unnecessarily burden computational resources.

Moreover, current open‐source platforms only allow data access to one user at a time. This may limit efficient use of clinical resources. To address these limitations, a SQL (Structured Query Language) database can be employed for data storage. With regard to size restrictions, the entire database is only restricted by the size of the available disk space on the computer or server hosting the database. More importantly, the SQL database also allows multiuser interaction simultaneously, which may improve efficiency of clinical resource usage. In essence, the SQL database is a fast and lightweight data storage system that can access only the queried data, without accessing large individual patient files in entirety. Because of these benefits, the standard for scalable databases is SQL. While there are SQL‐based software programs intended to create a database of DICOM data, such as DICOM Data Warehouse, those currently available are not specific to radiation oncology nor provide statistical analyses, to the best of our knowledge.^6^ Therefore, the aim of the proposed software is to provide an open‐source platform with a long‐term, scalable database and the statistical tools needed to explore and analyze a large set of data in a radiation oncology setting.

In the following, we develop a database and analytics platform that directly reads DICOM files and stores the parsed data in a SQL database. This tool provides visualizations and evaluation metrics for individual and multiple patients, with the aim to significantly reduce importing times for plans, while providing an overall clinical perspective of a large dataset. In Section 2, we discuss the data assumptions, database design, back‐end computations of additional anatomical metrics, as well as front‐end computations of plan evaluation metrics. Subsequently in Section 3, we present the results as a demonstration of the viability of the tool using an example dataset and not as actual clinical findings.

## 2 MATERIALS AND METHODS

**Figure 1:**
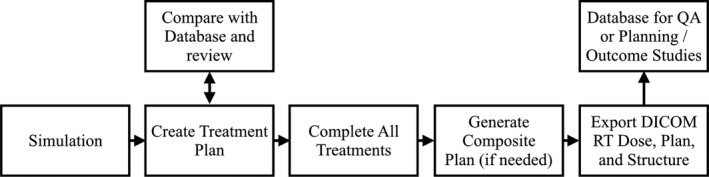
We propose this workflow as an example incorporating a DVH Database into a radiation oncology clinic. The bottom row represents a typical workflow without the use of a DVH database, while the top row indicates which stages a DVH database may provide benefit.

A database of DVH and treatment planning data can provide insightful information at the start and after the completion of a course of radiation treatment. Figure 1 illustrates a proposed workflow including such a database into a radiation oncology clinic. Combined with clinical outcome data, a DVH database can significantly reduce data collection time and potential transcription errors when correlating dosimetric data to patient outcomes. Alternatively, the combination of DVH and treatment planning data can be used to detect anomalous data during the final chart check prior to the first day of treatment, which may have otherwise gone unnoticed. Lastly, historical DVH data can be used for comparison to potentially indicate how a draft plan can be improved at the beginning of the treatment planning process. DVH Analytics was designed to facilitate these examples.

DVH Analytics is built in Python 2.7 using several open‐source libraries: pydicom to parse DICOM files, dicompyler to calculate DVHs from DICOM files, Shapely for polygon arithmetic, SciPy for statistics and fast OAR to planning target volume (PTV) distances calculations, Statsmodels for multivariable regressions, psycopg2 to communicate with a PostgreSQL database, and Bokeh to generate a web‐based user interface to query, plot, and administer the database.^7^, ^8^, ^9^, ^10^, ^11^, ^12^, ^13^, ^14^ All of these choices were made in the interest of ease of development and flexibility of deployment. As such, DVH Analytics is available for free use on all three major desktop operating systems (i.e., Microsoft Windows, Mac OS, and Linux). Although not a strict requirement, it is recommended to install a DICOM listener on the computer or server with DVH Analytics or have network access to a DICOM directory available to the user's treatment planning system. In this study, we use DCMTK DICOM Server as it is open source.^15^ However, DVH Analytics only needs operating system access to a file directory containing the files to be imported; it is up to the user to decide how to get the DICOM data into the directory.

### 2.A Assumptions

The initial intent of this database was to capture treatment planning DVHs in a clean and easily searchable manner. It is expected that there is only one complete, composite dataset per course of treatment, including all boosts. That said, each prescription will be included in the prescription, plan, and beam data, but the DVH data will represent only the final composite treatment plan for each structure.

Furthermore, the software was designed to automate data collection by extracting all required data directly from DICOM files. In this study, we validated the import process on six different treatment planning systems: Philips Pinnacle^3^ 8.0 m through 9.10, Elekta Monaco 5.0, Brainlab iPlan 4.5, Raysearch Raystation 5.0 through 6.1, Oncentra Brachytherapy 4.5, and Varian Eclipse 10. Currently, the only planning system among this group that does not explicitly include prescription information in the DICOM files is Pinnacle^3^. Therefore, a Pinnacle^3^ Script was generated to record prescription information into the names of points of interest (POIs) within the DICOM RT Structure file.

DVH Analytics provides a method to generate a region‐of‐interest (ROI) map for each physician. Although ROI categorization happens at the time of import, an admin user can initiate reprocessing without the need to reimport DICOM files. With only these three exceptions (i.e., prescription, treatment site, and ROI categorization), all other data are extracted from DICOM files without additional user input. However, it still is possible for some data to be incorrect or missing (e.g., physician, patient date of birth, simulation study date). Therefore, DVH Analytics provides an administrator view to query and edit any parameter after import. For users familiar with PostgreSQL, the database could be managed from command line or any PostgreSQL compatible database management software.

### 2.B SQL database design

The primary data for DVH Analytics is organized into four tables: DVHs, Plans, Prescriptions, and Beams. These tables are linked by patient medical record number (MRN) and study instance unique identification (UID). The contents of these tables are listed in Table 1. The MRN is extracted from the “Patient ID” tag in the DICOM RT files. The study instance UID is the same parameter used by DICOM to uniquely identify all files associated with a particular image study (e.g., CT simulation). Both the DICOM RT Dose and DICOM RT Structure files are required to extract or compute this DVH data.

**Table 1:** SQL table design for DVH Analytics

DVHs	Plans	Prescriptions	Beams
MRN	MRN	MRN	MRN
Study instance UID	Study instance UID	Study instance UID	Study instance UID
Import timestamp	Import timestamp	Import timestamp	Import timestamp
DVH	Age at study date	Fractions (Fxs)	Beam name
PTV distance	Baseline	Fx dose	Beam number
PTV overlap	Birthdate	Fx group name	Beam dose
ROI name	Dose grid resolution	Fx group number	Beam MU
ROI type	Dose timestamp	Fx group count	Beam MU per degree
ROI coordinates	Fractions	Norm. method	Beam MU per control point
ROI dose	Heterogeneity correction	Norm. object	Beam control point count
ROI institutional category	MU (plan total)	Prescription dose	Beam energy (min/max)
ROI physician category	Patient sex	Prescription percent	Beam radiation type
ROI volume	Patient orientation		Beam type
	Physician		Collimator angle information
	Plan timestamp		Couch angle information
	Prescription dose		Fx count
	Sim study date		Fx group beam count
	Structure timestamp		Fx group number
	TPS manufacturer		Gantry angle information
	TPS software name		Isocenter
	TPS software version		Scan spot count
	Treatment modality		Scan mode
	Treatment site		SSD (Avg SSD for arcs)
	Treatment time		Treatment machine

The SQL database for DVH Analytics was designed to include the following data. Each column of this table represents a unique SQL table with SQL table's columns named based on the data listed here. Each of the SQL tables are linked by MRN and study instance UID.

The data in the Plans table includes patient demographics (i.e., age, birthdate, and gender), physician's initials, patient's treatment orientation, treatment planning system data (i.e., manufacturer, software version, heterogeneity corrections, and dose grid resolution), treatment modality (e.g., Photon arc, Protons, Brachytherapy), and treatment time (for Brachytherapy). DICOM RT Dose, Plan, and Structure files are required to build this table. The treatment site defaults to the plan name. However, if the user creates a POI in the TPS, prior to export, beginning with “tx:,” DVH Analytics will set the treatment site to the text that follows. For example, a POI name of “tx: Brain” will prompt DVH Analytics to set the treatment site to “Brain.” This method is similar to that used to extract prescription information for plans exported from Pinnacle^3^.

The Prescriptions (Rxs) table contains the fraction group data. Aside from the plan name, which is the same for each prescription, all data refer to the particular fraction group (e.g., initial, boost1, boost2, etc.). The Beams table primarily contains data specific to each beam, including beam energy min/max (proton beams have a range of energies), beam type (e.g., static, dynamic), scan spot count for proton plans, and gantry/collimator/couch information (i.e., start, stop, min, max, and range). Currently, all data in this table are applicable for linac or proton‐based treatments. Importing data into DVH Analytics from brachytherapy plans will not result in Beams table data; however, all other SQL tables will be populated.

Finally, a catalog of imported DICOM files is maintained using a fifth SQL table. This table includes MRN, study instance UID, the postimport directory which contains the DICOM files, and the file names of the RT Plan, RT Structure, and RT Dose files used for import. If multiple instances of a DICOM file type are found, only the file with the latest timestamp will be used for import; however, all files with the same study instance UID will be collected into the user‐specified import directory and further organized by the MRN. This feature allows DVH Analytics the ability to easily reimport data directly from DICOM files; it also allows for future development of DVH Analytics that may rely on imaging data.

### 2.C Back‐end computations

For the most part, there are explicit DICOM tags for the data contained in the DVH Analytics database. As DVH data often are not explicitly stored in DICOM files, DVH Analytics uses the dicompyler Python code to compute the DVH data.^8^ In addition, DVH Analytics stores information for the purposes of ROI name management and computes the union of all PTVs for calculations determining minimum ROI‐to‐PTV distances and PTV overlap. Anatomical factors, such as distance between structures, can have a significant impact on treatment planning goals. In fact, factors such as distance between PTV and a surrounding OAR, as well as the volume overlap between PTV and OAR, have been identified as significant predictors of DVH goals.^16^ In addition, radiobiological calculations are performed based on equivalent uniform dose (EUD) as described by Niemierko.^17^

#### 2.C.1 ROI name management

One of the more difficult challenges of maintaining a meaningful DVH database is overcoming variations in ROI names. For example, a particular physician or planner may choose to name the left eye any of the following: L Eye, Orbit left, eye l, etc. The only certain way to catch all ROIs intended to be the left eye is to always name the ROI the same exact way. In practice, this does not happen when the treatment planning software allows the user to type in any ROI name. As a way to mitigate this, DVH Analytics provides a method to map any number of possible ROI name variations. Over time and with user input, this system becomes more robust, reducing the likelihood of missing a ROI categorization. From the Admin view, a user can view a list of all uncategorized ROIs. From this list, the user may tag the ROI as “ignore” so that it is removed from the list or add the ROI to the ROI map.

**Figure 2:**
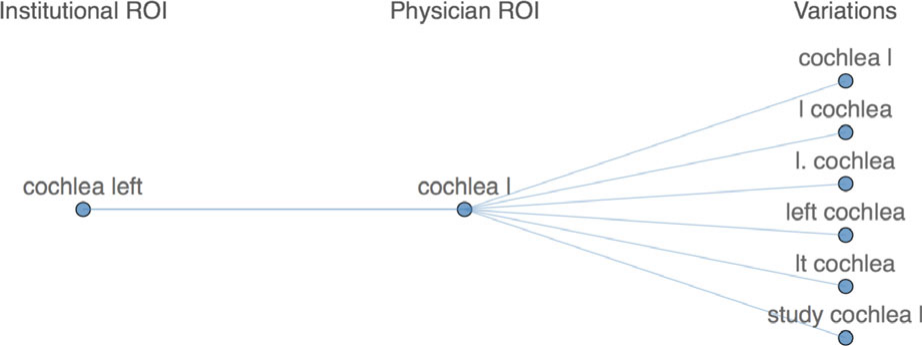
Screenshot from the ROI Name Manager module in DVH Analytics illustrating possible variations for the left cochlea. When a plan is imported for the associated physician, any ROI name matching one of the variations will automatically be mapped to the corresponding Physician ROI and Institutional ROI. Additional details are in Section 2.C.1.

This mapping system uses two separate ROI name categories: institutional ROI and physician ROI. Institutional ROIs are names used to define a sample of DVHs across the entire database, whereas a physician ROI is curated for a particular physician's practice, which will either map to an institutional ROI or be left as uncategorized. This allows physicians the flexibility to create their own naming system as well as the ability to track more anatomically specific ROIs for their specialty. DVH Analytics provides a view of any selected ROI name as shown in Fig. 2, which illustrates another example of potential variations for the left cochlea.

For tumor/target volumes (e.g., gross tumor volume (GTV), clinical target volume (CTV), and PTV), DVH Analytics records the DICOM information containing the structure type (e.g., PTV, Organ at Risk, External, etc.). It is recommended that these tags be appropriately defined prior to DICOM export. For plans with multiple PTVs, DVH Analytics will assume a naming scheme of PTV1, PTV2, PTV3, etc., ordered by the D_95%_ of each volume. However, the user could choose to create a consistent ROI naming scheme and add this information into the ROI Name Manager for greater flexibility. Currently, some planning systems do not export DICOM tags that include internal target volume (ITV) as a structure type (e.g., versions of Pinnacle^3^ at least 9.10 and earlier). However, DVH Analytics will tag any structure that begins with “ITV” as such in the database, regardless of the associated structure type in the DICOM file.

#### 2.C.2 Geometric computations

DVH Analytics provides a method to calculate the geometrical union of ROIs; this method is specifically applied to generate a combined PTV for the purposes of computing ROI distances to the combined PTV as well as PTV overlap. For convenience and computational efficiency, we employ the Python packages Shapely and SciPy.^9^, ^10^

Shapely provides a convenient way to perform geometric operations between two‐dimensional polygons, specifically the calculation of intersections, differences, and unions. After converting the DICOM coordinates of a ROI into ordered sets of points, polygons representing the ROI are generated with Shapely. Per DICOM convention, if multiple polygons exist in a single slice (2D image), a subsequent polygon that exists inside the cumulatively generated polygon represents a subtraction of area from the cumulatively generated polygon (e.g., a ring structure). Likewise, a subsequent polygon outside the cumulatively generated polygon represents an island structure (e.g., delineating both left and right lungs in a single ROI). With this understanding, the authors generated a combined polygon (i.e., a MultiPolygon class in the Shapely code) for the PTV, and separately, the OAR, accounting for any holes or islands.

##### PTV union

All calculations in this section are based on a combined PTV. The method employed to compute the combined PTV is as follows. First, the coordinates of all ROIs with the ROI types beginning with PTV are retrieved from the SQL database. Second, the coordinates from each slice (or z‐coordinate) of each ROI are converted into polygons with Shapely. The union of all polygons of a given slice is calculated, and the coordinates of the subsequent polygon of every slice are then stored. This method returns a complete 3D geometric union of all PTVs in the same format as any other ROI in DVH Analytics.

##### PTV overlap

After the generation of the combined PTV, the intersection of the resulting MultiPolygon with the ROI is calculated for each slice. The resulting areas of these intersections are multiplied by their respective slice thickness. Then, these volumes are summed to calculate the PTV overlap volume. The slice thicknesses are obtained from the z‐coordinates of the slice of interest and an adjacent slice.

##### Minimum ROI to PTV distances

A brute‐force method of calculating all distances between points defining the PTV surface to all points defining the ROI surface is employed to compute the minimum ROI to PTV distance. The minimum distance between the PTV and the ROI is the minimum of all the distances computed. DVH Analytics also records the mean, median, and maximum of these distances to provide additional spatial context. This brute‐force method can be very computationally expensive, particularly with straightforward methods of lists and for‐loops in Python. To overcome this limitation, we employ the SciPy library, which includes a “spatial” module with a function to do these distance computations.^10^ We observed more than an order of magnitude reduction in computation time compared with non‐library‐dependent methods in Python. This results in computation times on the order of 100 ms for most ROIs, which is relatively small compared with the DVH calculation times. Additional computation times are reported in Section 2.E. PTV distances to large ROIs (e.g., external, skin) could be calculated in under a second; however, memory issues could arise. Therefore, only categorized ROIs that are not external or skin are calculated by default. However, the user may manually trigger this calculation for specific ROIs in the admin view of DVH Analytics.

#### 2.C.3 Radiobiological metrics

EUD, tumor control probability (TCP), and normal tissue complication probability (NCTP) are computed with the DVHs calculated by dicompyler, using the formalism described by Niemierko.^8^, ^17^, ^18^ For convenience, several published values by Emami et al. for multiple OARs are provided in the Rad Bio module of DVH Analytics; options are given that the user may choose to apply their own values for the biological calculations.^19^ Once calculated, the user may include EUD, TCP, and NTCP values in the Time‐Series, Correlations, and Regression modules, as discussed later in Sections 2.D.4. and 2.D.5.

### 2.D Main application view

The main application view is split into eight tabs: Query, DVHs, Rad Bio, ROI Viewer, Planning Data, Time‐Series, Correlation, and Regression. The contexts of these tabs are described in the following subsections.

#### 2.D.1 Query

The initial view of DVH Analytics is a module for the user to design their query. As opposed to requiring the user to learn command‐line syntax of PostgreSQL, DVH Analytics provides a series of dropdown menus grouped by “Selection Filters” and “Range Filters,” which prompt the user for discrete and non‐discrete data constraints, respectively.^12^ These categories are listed in Table 2. Once a Selection Filter category from Table 2 is selected, the adjacent dropdown will automatically populate with all choices available in the user's database. Likewise, once a Range Filter category is assigned, the titles for the two adjacent input fields for minimum and maximum values will update based on the user's database. Any of these categories can be combined with any number of definitions to generate the final query. If two identical categories are defined within the same Group (Blue or Red), the query will assume an “or” operator.

**Table 2:** Searchable categories

Selection filters	Range filters
Baseline	Age (at study date)
Beam type	Beam dose
Collimator rotation	Beam energy
Couch rotation	Beam MU
Dose grid resolution	Birthdate
Gantry rotation	Collimator angle
Heterogeneity correction	Couch angle
MRN	Distance to PTV
Norm. method	Fraction dose
Patient orientation	Gantry angle
Patient sex	Planned fractions
Physician	ROI min/mean/max dose
ROI institutional category	ROI volume
ROI physician category	Rx dose
ROI type	Rx isodose
Radiation type	Scan spots (protons)
Scan mode (protons)	Simulation date
Treatment machine name	SSD (Linac)
Treatment modality	Total plan MU
Treatment site	Treatment time (brachy)

The categories listed in this table are available for query definitions in DVH Analytics.

**Figure 3:**
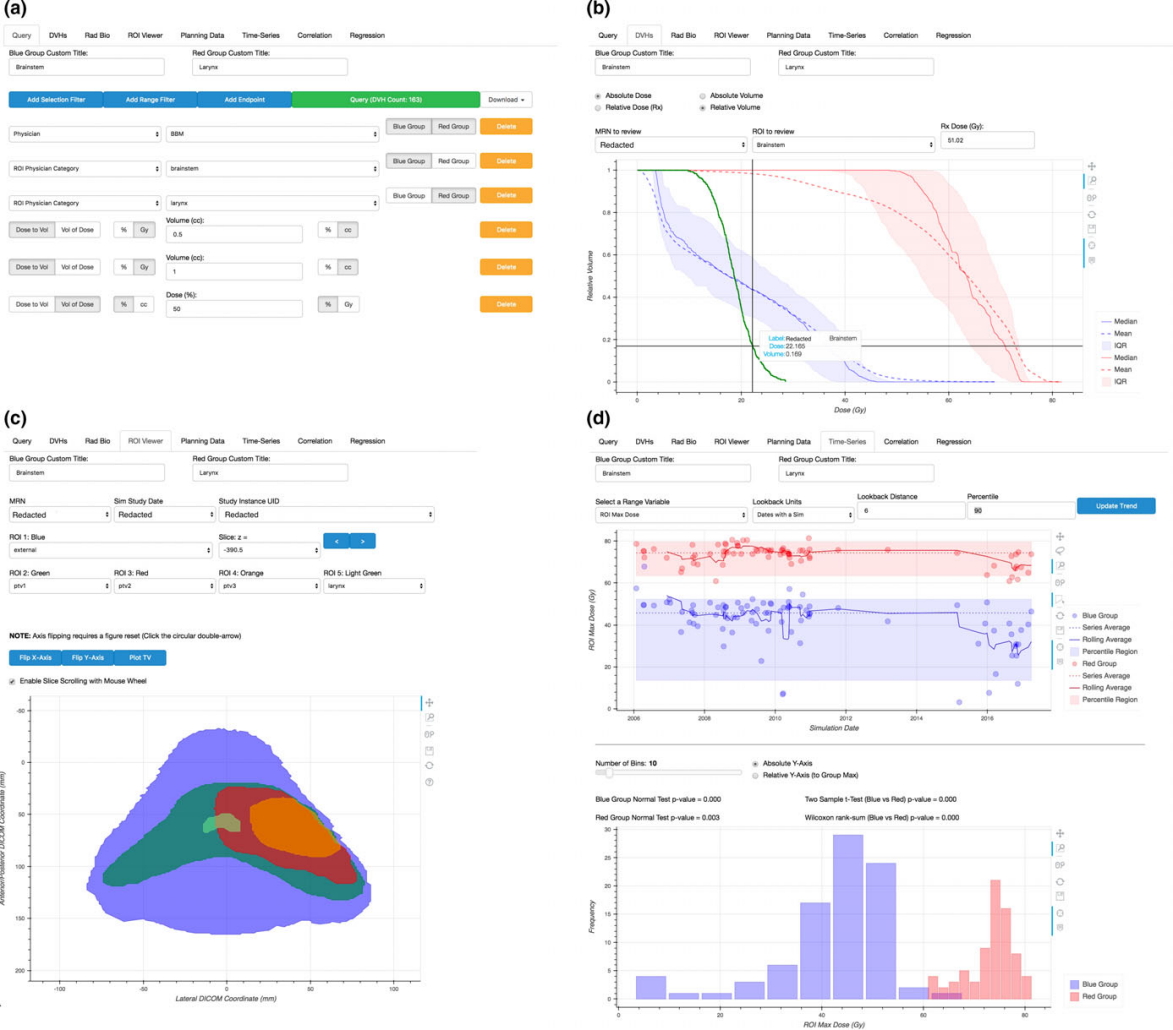
Example views of Query, DVHs, ROI Viewer, and Time‐Series tabs within DVH Analytics. (a) Queries are generated with a series of dropdown menus populated by existing data within the user's SQL database. Text input is required to filter continuous data or define DVH endpoints. All data in subsequent tabs are based on the filters defined here. (b) Interquartile, mean, and median DVHs are shown by default, but all DVH data can be displayed in this plot by selecting DVHs in the table below (not shown). One DVH from the “review” folder may be plotted here as well. (c) The ROI Viewer can plot any ROI from the database; however, only MRNs from the queried sample are selectable. (d) The Time‐Series tab presents time‐series plots, their respective histograms, as well as *P*‐values from normality tests, two‐sample *t* test, and Wilcoxon rank‐sum test.

In addition, the user may define up to eight “Endpoints” to be tabulated. Each endpoint added allows the user to define a dosimetric (e.g., D_2 cc_, D_95%_) or volumetric (e.g., V_20%_, V_50Gy_) point for all DVHs in the query. These values can be reported in absolute units of cm^3^ or Gy, as well as in a relative scale (relative to volume or prescription dose), as shown in Fig. 3(a).

Once the user has defined the desired sample based on any number of Range or Selection Filters, clicking the update button will retrieve all data stored in the database that fits the query. All information presented in the remaining tabs is based on this retrieval.

#### 2.D.2 DVHs and planning data

DVH Analytics provides an interactive plot containing up to two separate interquartile ranges (IQRs) of user‐defined DVH samples as well as the option to plot a single DVH from DICOM files located within the user‐defined “review” directory, as shown in Fig. 3(b). The reviewed DVH is not included in the sample statistics calculations.

The plot will display the interquartile range, mean, and median DVH of each defined sample. Each of these computations are performed by calculating the appropriate percentile or mean of each dose bin across all queried DVHs. A table displayed below the DVH plot reports the patient's MRN, ROI name and type, the composite plan's total prescription dose, ROI volume, the ROI's min/mean/max dose, and the minimum distance to PTV, and PTV overlap. A second table is populated by the DVH endpoints defined by the user. The Planning Data tab displays all of the prescription, plan, and beam data for the query.

#### 2.D.3 ROI viewer

DVH Analytics provides a visualization of ROIs from a specified study instance UID (as filtered from the query and, subsequently, MRN and study date); this is illustrated in Fig. 3(c). This module processes the DICOM coordinates of the specified ROIs into polygons, which may be viewed two dimensionally, one slice at a time.

#### 2.D.4 Time‐series plots

A time‐series plot to demonstrate trends across simulation dates are provided in the Time‐Series tab, as illustrated in Fig. 3(d). The *y*‐axis of this time‐series plot may include a EUD, TCP, NTCP, a DVH endpoint, or any of the Range Filter variables listed in Table 2. A box is shown on the plot indicating the user‐specified percentile bounds of the sample, and the sample mean is displayed as a horizontal line spanning all dates within the sample. In addition, a moving average is plotted with a user‐defined look‐back window. Each point on the moving‐average line represents the mean of all data from the point's date ranging back until the user‐defined length of time. To exclude any data from being considered for the computation of the moving average, we rely on the “lasso select tool” developed by Bokeh, which allows users to draw a shape such that all points within the shape will be selected. Then, we exclude the selected data from the moving‐average computation and plotting.

#### 2.D.5 Tests of means, correlations, and regressions

DVH Analytics provides three analytical modules: testing of sample mean differences, Correlations, and Regressions. A two‐sample *t* test on the difference of means between the two selected groups of data is conducted and a *P*‐value is reported. In addition, for nonparametric data, a Wilcoxon rank‐sum test is provided with the p‐value. The Correlations module generates a correlation matrix of many of the continuous variables listed in Table 2, user‐specified DVH endpoints, or computed radiobiological values (i.e. EUD, TCP, NTCP). The Pearson‐*R* value for each variable pair is represented by a color‐coded circle, which has a radius and opacity that scales with the magnitude of the Pearson‐*R* value, as calculated with the stats module of SciPy.^10^ This provides the user with a clear visual indicating strong or weak correlations. The information provided in the correlation matrix may help guide the user's attention to likely relevant variables for predictive modeling in the Regressions module. In the Regressions module, the user may plot any of the variables listed in the Correlations module against another. Along with this plot, the results of a univariable linear regression are displayed in a table including slope, y‐intercept, *R* ^2^, *P*‐value, standard error, and sample size, as calculated by the stats module of Scipy. The user may tag any of these variables to be included in a multiple linear regression conducted using the Python package Statsmodels.^11^ The results of this multiple regression are displayed in a table including the *R* ^2^ and probability for the F‐statistic of the regression as well as the coefficient and *P*‐value for each tagged variable.

### 2.E Importing and processing times

DICOM file importing times were tested on a 2016 MacBook Pro running MacOS Sierra (10.12.6) with a 2.6 GHz Intel Core i7 and 16 GB of RAM. For testing purposes, a sample of 70 clinically used plans were imported. The total import time was 42 min 35 s, resulting in an average import time of 37 s per plan. The total time taken to compute all PTV distances to the 2,063 categorized ROIs was 26 min 35 s, resulting in an average computation time of 0.8 s per ROI; the total time to compute the PTV overlap volumes in 3,273 ROIs was 26 min 45 s, resulting in an average computation time of 0.5 s per ROI.

## 3 RESULTS

### 3.A Data validation

**Figure 4:**
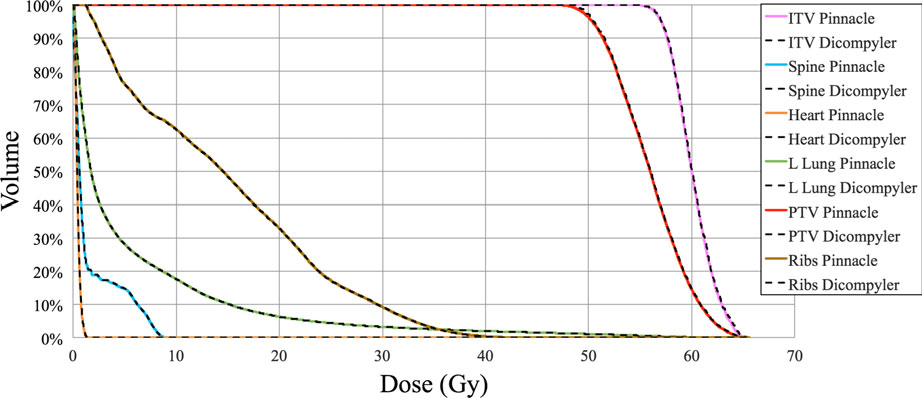
DVH comparison of calculations from DVH Analytics (computed with dicompyler) and Pinnacle^3^.

DVH calculations were validated against DVH data extracted from Pinnacle^3^ using a script that stores the data into an ASCII file. ROI volumes and selected DVH points were manually recorded as displayed in Pinnacle^3^. All DVH Analytics data were extracted from the csv file generated when clicking the “download” button in the DVH Analytics application. These data were collected into a spreadsheet for plotting and tabulation to ensure independent validation DVH Analytics with another DVH calculation method. DVHs for ITV, PTV, spine, left lung, heart, spine, and ribs were plotted, as shown in Fig. 4. The DVHs are virtually identical, with the largest deviation on the initial shoulder of the PTV.

The calculations for ROI volume, PTV overlap, and minimum distance from ROI to PTV were validated by comparing these results to those computed in Pinnacle^3^ with ROIs from a randomly selected plan (nonanatomical ROIs were omitted for brevity, e.g., ROIs for optimization). These data are reported in Tables 1, 2, 3, located in the Appendix.

Volume calculations are performed with the code from dicompyler.^8^ The data in Table A1 show a maximum absolute difference of 2.76 cm^3^. All absolute differences greater than 0.5 cm^3^ correspond to relative differences of typically 1–3%, at most 6.2%. Because dicompyler's volume calculation is dependent on the dose grid resolution (at the time of this study), these differences can be reduced further by calculating the dose grid with a finer resolution prior to DICOM export from the treatment planning system. The values reported in Table A1 are based on a 4‐mm cubic dose grid resolution. These deviations are comparable to other studies reporting on variations of volumes between multiple treatment planning systems.^20^, ^21^

The 40 ROIs reported for volume calculation comparisons were also used to validate PTV overlap calculations in DVH Analytics against those calculated with Pinnacle^3^. Pinnacle^3^ does not directly report PTV overlap. Therefore, PTV overlap calculations are performed using Pinnacle^3^'s ROI Expansion/Contraction tool. First, the union of all PTVs was calculated and stored into a separate ROI. Then, the intersection of this combined PTV with each remaining ROI was generated one at a time. The volume of these subsequent ROIs, as calculated by Pinnacle^3^, is reported as PTV overlap in Table A2. Only two calculations resulted in an absolute difference greater than 0.21 cm^3^; both of these calculations were for large structures (i.e., external and skin).

The minimum distance to PTV for the same ROIs used in Tables A1 and A2 was calculated as described in Section 2.C.2, with the exceptions of external and skin ROIs as these are omitted in DVH Analytics due to potential memory overload issues. Although Pinnacle^3^ does not explicitly calculate distances between ROIs, Pinnacle^3^ does have functionality to calculate distances between two user‐specified points. When possible, the distances reported in Table A3 are the minimum distance measured in any of the orthogonal planes (axial, sagittal, or coronal) of several measurements. In the few cases when the listed ROI could not be viewed in an axial, sagittal, or coronal plane with the PTV simultaneously, points were placed in the nearest corners of the ROI and PTV; the 3D distance between these two points was reported. Notably, all absolute differences greater than 2 mm correspond to relatively small ROIs comprising a small number of slices. Considering the plan selected for this analysis is based on a CT study with 3‐mm thick slices, an absolute difference of 3‐mm perpendicular to axial planes is not unexpected. The largest absolute difference was 3.4 mm.

### 3.B Data exploration

While the authors’ initial intent for the design of DVH Analytics was to develop a queryable database of DVHs, the collection of a large subset of the DICOM data other than DVHs originally meant for query definitions has added significant value. Simply plotting any of these data over time can reveal potentially invalid data, quality control metrics, or temporal variations (e.g., across a physician, the institution, treatment site, etc.).

#### 3.B.1 Seeking “Bad” data

The benefit of time‐series plots is not exclusively individual patient QA or easy data aggregation for clinical outcome studies; time‐series plots also provide a valuable method for seeking incorrect or incorrectly categorized data. For example, when plotting the H&N larynx volume data, it was observed that one larynx volume was more than double the average of the sample. Upon inspection, the outlier was actually due to the incorrect categorization of the ROI. Ostensibly, the name of the ROI was a misspelling of larynx. In fact, the ROI was poorly labeled; it was an expansion from the anatomically delineated ROI and used for planning purposes. This is clear evidence that automated categorization of ROIs is not without caveats and should serve as a warning to users. The authors recommend plotting and examining various variables in this fashion after importing new data as a time‐series plot can easily demonstrate gross outliers.

#### 3.B.2 Quality control metrics with context

**Figure 5:**
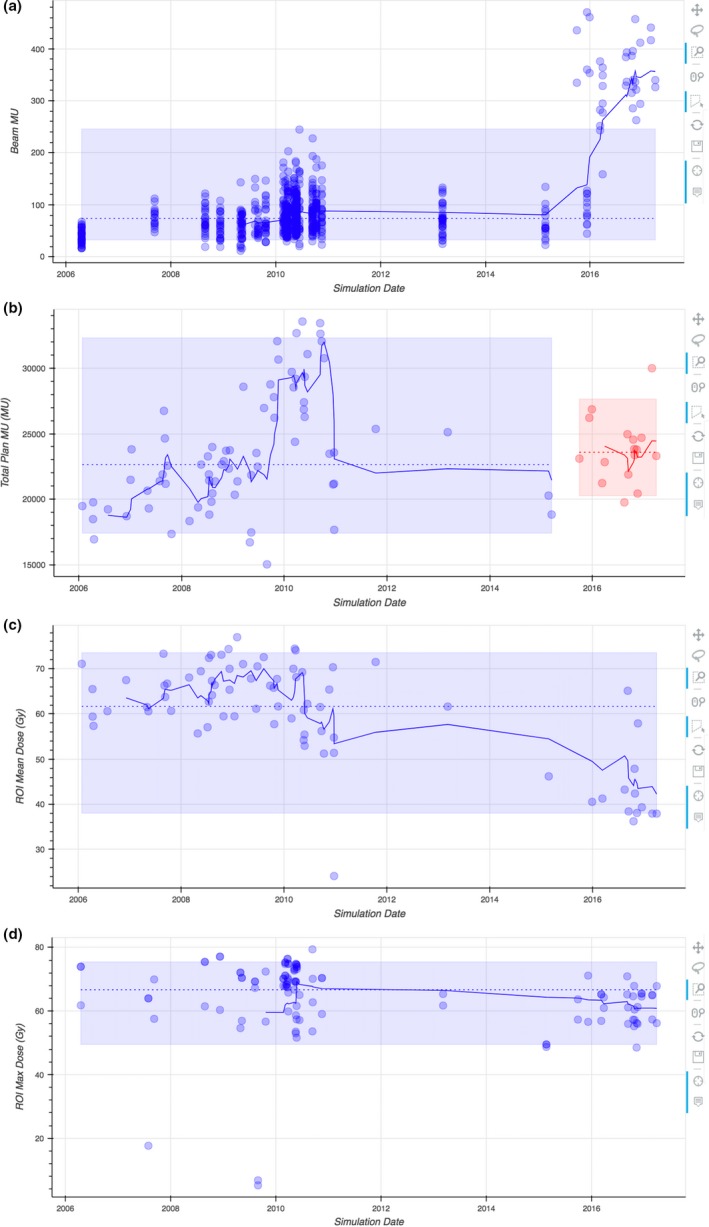
Example time‐series plots using H&N planning data: (a) beam MU, (b) total plan MU of IMRT (blue/earlier) and VMAT (red/later) plans, (c) mean larynx doses, and (d) maximum brachial plexus doses for a single physician. In all four plots, the shaded regions contain 90% of the data within the respective sample, the dashed lines represent the sample mean, and the solid lines represent a rolling average with a look‐back window of 6 simulation dates.

Observing temporal changes in data can provide valuable insight to build physician‐specific profiles of typical dose constraints, indicate plans with atypical parameters, and even help correlate patient toxicities to dosimetric data. For example, Fig. 5(a) illustrates a change in beam (MU) of 75 plans spanning 11 years. All of these plans are H&N plans from a single physician in a single institution. All plans were generated using Pinnacle^3^ and planned with step‐and‐shoot IMRT or VMAT delivery techniques. Figure 5(a) illustrates a significant increase in beam MU over time. Furthermore, Fig. 5(b) provides context about the clinic in that treatment techniques transitioned from step‐and‐shoot IMRT to VMAT arc deliveries. Because of the treatment technique change, planning parameters derived from beam MU should not span both IMRT and VMAT datasets. Instead, they should be derived by treating IMRT and VMAT as separate samples. The important takeaway from Fig. 5(b) is that examination of an outlier should consider multiple variables and include clinical context. Therefore, DVH Analytics provides this quality check with emphasis to examine each outlier.

The data in Fig. 5(c) illustrate a decrease in the mean larynx dose of H&N plans from a single physician's practice over the span of about 11 yr. The institution transitioned to VMAT delivery around the start of 2016. It would appear as though VMAT deliveries generally result in lower mean larynx doses. However, this change is reflective of a change in planning choices and physician practice. Likewise, Fig. 5(d) shows a similar trend for brachial plexus doses. Again, the downward change is more indicative of physician preference than technological capabilities. Notably, there are three clear outliers in Fig. 5(d). Upon inspection, the authors discovered that these three points can be explained by correlating the ROI minimum distance to PTV. A series of plots such as these could provide a planner enough information about what a physician typically expects and drive the plan toward the physician's preferences and/or clinical norm.

#### 3.C Data analysis

**Figure 6:**
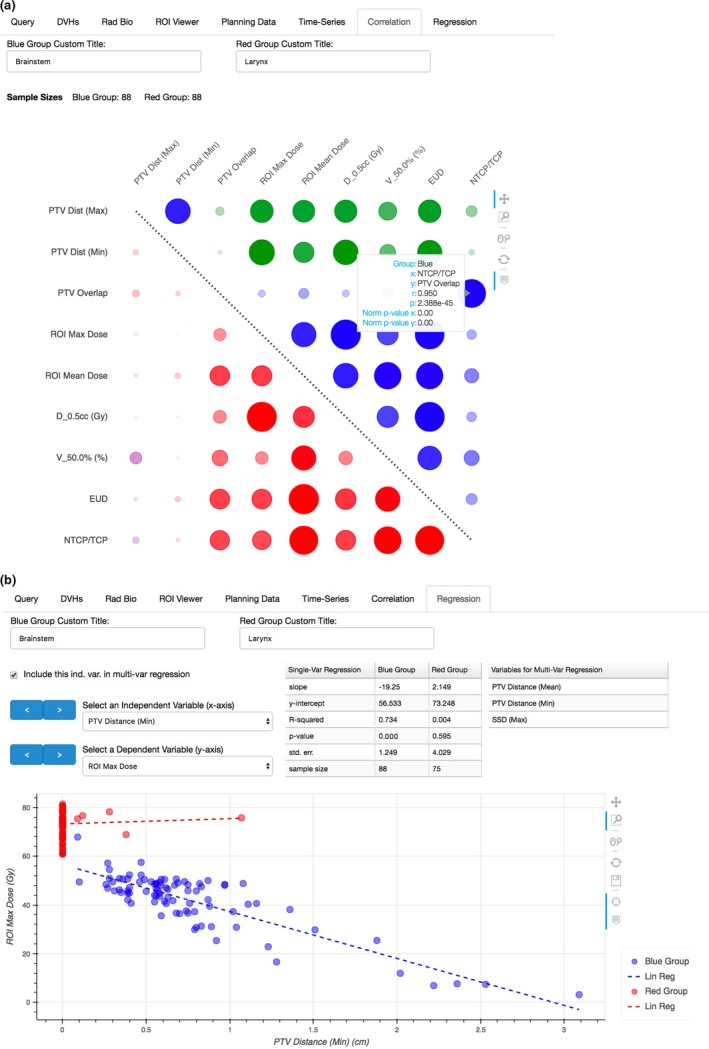
A correlation matrix and linear regression of brainstem and larynx data from H&N plans. (a) The top right of the correlation matrix represents brainstem data (blue group), while the bottom left represent larynx data (red group). The diameter and opacity of each circle scale with the magnitude of the Pearson‐*R* correlation. Green and purple colors indicate negative correlations for brainstem and larynx data, respectively. (b) Univariable linear regression parameters are reported for both brainstem and larynx data. After inspection, the user may indicate which variables are to be included in a multivariable regression. The results of the multivariable regression with this brainstem data are tabulated in Table 3.

Pearson‐R correlations between nine variables with a dataset of 88 patients are presented in Fig. 6(a) for brainstem and larynx data. Some of the data points in this matrix have strong correlations but have relatively little utility in a clinical setting. For example, a strong correlation between the mean and median PTV distances is more indicative of the skewness of the dataset. However, the correlation values observed between PTV distance and ROI dose are of interest. In the case of brainstem, being a serial structure, the correlation values observed between PTV distance and ROI dose may be of clinical significance in evaluating plan quality and/or OAR constraints. In this H&N dataset, the larynx mostly overlaps with the PTV, and hence, little correlation is observed. However, the brainstem never overlaps resulting in a strong negative correlation.

Multivariable linear regressions were performed, with the same dataset used to generate the correlation matrix in Fig. 6(a), using Statsmodels.^11^ These results are tabulated in Table 3. The models indicate a significant correlation between the minimum and average PTV‐to‐brainstem distances and the maximum brainstem dose, which is consistent with the previously discussed correlation matrix. Interestingly, Model 1 summarized in Table 3 indicates a strong correlation with the maximum source‐to‐skin distance (SSD) of the treatment beams. However, it is important to appreciate that there is likely a more fundamental independent variable at play than SSD (e.g., laterality of PTV or beam isocenter, patient weight or size, etc.). Model 2 also is significant, but reports a reduction in correlation. The same two models applied to larynx data reported poor correlation (i.e., *R* ^2^ = 0.316 and *R* ^2^ = 0.038, respectively), demonstrating the need for context as these regression models for brainstem data are clearly not suitable for the larynx data.

**Table 3:** Two multivariable models generated with DVH Analytics

Regression model	Independent variable	Coefficient	*P*‐value
Model 1 (*R* ^*2*^ = 0.819, *P* = 0.000)	Constant	−80.62	0.005
	PTV distance (mean)	−6.932	0.000
	PTV distance (min)	−8.028	0.005
	SSD (max)	1.547	0.000
Model 2 (*R* ^*2*^ * *= 0.760, *P* = 0.000)	Constant	63.74	0.000
	PTV distance (mean)	−5.586	0.003
	PTV distance (min)	−10.58	0.001

Results from multivariable regressions for the maximum brainstem doses contained within the data used to generate Figs. 6(a) and 6(b).

## 4 DISCUSSION

As with any database, maintaining its integrity is critical for useful implementation. There are a number of points‐of‐failure that DVH Analytics is not equipped to handle in an automated fashion. The methods below are suggestions from the authors to help mitigate these issues.

### 4.A Planning vs anatomical structures

Arguably, the biggest flaw in a DVH database is that much of its data are based on subjective delineation of anatomical structures. Furthermore, although with the best of intentions, it is not uncommon for physicians or treatment planners to exaggerate/expand these delineations in the interest of patient safety, with respect to evaluating dose constraints. Strictly speaking, these exaggerated/expanded OARs should be denoted as PRVs (Planned Risk Volumes); however, this is not always done in practice. Therefore, users who wish to implement DVH Analytics should be aware of these differences. However, users wishing to use a database such as DVH Analytics for the purpose of seeking a correlation between dosimetric data and patient toxicities should strongly consider careful ROI delineation with appropriately named ROIs and ROI mappings before drawing any conclusions.

### 4.B Database gatekeeper

Although DVH Analytics provides automation methods for parsing data into a database, there is still some manual effort required by the user to maintain the integrity of the data quality. For instance, the source of some of the data included in DICOM files can be from manual entry (e.g., date of birth, MRN, gender, sim study date). It is worthwhile to implement a second check to verify the DICOM data for these tags against patient records at the time of import. Although DVH Analytics technically is capable or importing multiple plans for a single study instance UID (or CT‐sim study), the automated import process does not allow this to occur, as a means to notify the user of potential data duplication. As such, the authors recommend users to wait until a patient has fully completed his or her course of treatment to ensure the composite plan is imported into the database. It is also important to monitor which ROIs do not get categorized. After a large number of plans are imported for a particular physician, this need is reduced. However, manually updating a physician's ROI naming map from a large number of uncategorized ROIs may lead to categorization mistakes or unintentional and unnoticed deletion of otherwise useful DVH data. Appointing a “database gatekeeper” can help mitigate many of these issues and lead to a more consistent database quality. Someone with the experience of seeing all data entered into the database should have a better chance of spotting inconsistencies. This will better establish the utility of labeling a particular plan as “baseline” to be used for quality control purposes. The intent of this category is to allow a user to tag a plan as being suitable for building baseline statistics, which is entirely based on the user's judgment. For example, a user may not want to include a patient being treated with two lung tumors simultaneously to determine typical lung DVH endpoints.

### 4.C Treatment site and ROI naming policies

As with any database, drawing useful conclusions requires proper context. In the case of a DVH database, DVHs need to be grouped by treatment site and anatomy, at a minimum. For example, it does not make any sense to compare the lung DVH of a patient being treated with a lung tumor to that of a patient whose treatment was for a brain tumor. Including lung DVHs from all treatment sites will unnecessarily skew data and reduce the ability to determine outliers for the purposes of quality control. Therefore, it is important for users of a DVH database to decide on a consistent list of treatment sites. With respect to ROI name categorization, although DVH Analytics provides a way to build a ROI name map, consistent ROI naming will reduce the chance of leaving a ROI uncategorized or incorrectly categorized. In addition, the authors suggest a ROI naming policy that considers factors such as surgical status (i.e., pre‐ or postsurgery) for tumor volumes.

### 4.D Future research

Considering that radiation treatment is just one piece of cancer care for many patients, our next step is to seamlessly connect pertinent patient data from other treatment modalities. We are particularly interested in combining the DICOM data extraction and statistical tools currently available in DVH Analytics with information such as cancer staging, chemotherapy agents/prescriptions, surgical status, and clinical outcome data (including radiation induced toxicities).

## 5 CONCLUSION

A free and open‐source software application called DVH Analytics is developed that can store, organize, parse, and analyze non‐image‐based DICOM data for use in a radiation oncology setting. The software accepts DICOM files as input data, allowing a seamless transition between DVH Analytics and treatment planning systems. To highlight a few tools, the software allows time and memory efficient queries on single parameters of large patient datasets, displays ROIs, performs statistical tests, and builds predictive models using a database. DVH Analytics is available at http://www.dvhanalytics.com. Newer versions will be updated at the same location as new tools are developed.

## CONFLICT OF INTEREST

No conflicts of interest.

## APPENDIX 1

### 1.1

**Table A1:** Volume (cm^3^) calculation comparisons of dicompyler and Pinnacle^3^

ROI name	DVH Analytics	Pinnacle^3^	Abs. Diff.	Rel. Diff.
Base of tongue	28.90	29.28	0.38	1.30%
Brachial plexus total	18.53	18.58	0.05	0.27%
Brain	1285.06	1287.82	2.76	0.21%
Brainstem	21.84	21.69	0.15	0.69%
Cervico‐thoracic esophagus	17.23	17.85	0.62	3.47%
Cochlea l	0.14	0.25	0.11	40.00%
Cochlea r	0.14	0.18	0.04	22.22%
Constrictors total	20.3	21.64	1.34	6.19%
Ext	8430.77	8429.25	1.52	0.02%
Eye l	10.03	10.17	0.14	1.38%
Eye r	9.70	9.9	0.21	2.12%
Glottic larynx	10.75	10.46	0.29	2.77%
Inferior pharyngeal constrictors	3.02	2.77	0.26	9.39%
Larynx total	33.94	33.75	0.19	0.56%
Left brachial plexus	8.83	8.78	0.06	0.68%
Lips	3.70	3.63	0.06	1.65%
Mandible	54.91	54.62	0.29	0.53%
Middle pharyngeal constrictors	1.54	1.87	0.33	17.65%
Opticchiasm	1.01	1.18	0.17	14.41%
Opticnerve l	0.77	0.8	0.03	3.75%
Opticnerve r	0.82	0.77	0.05	6.49%
Oralcavity	66.67	65.49	1.18	1.80%
Parotid l	18.10	18.06	0.03	0.17%
Parotid r	16.13	16.28	0.15	0.92%
Parotid total	34.27	34.34	0.07	0.20%
Pituitary	0.24	0.42	0.18	42.86%
Posterior cricoid esophagus	1.06	1.23	0.17	13.82%
Presurgery gtv	12.38	12.18	0.21	1.72%
PTV 54 Gy	243.22	244.66	1.44	0.59%
PTV 56 Gy	69.74	69.87	0.13	0.19%
Right brachial plexus	9.65	9.81	0.16	1.63%
rt. neck bst PTV 6 Gy	21.26	20.74	0.52	2.51%
Skin	2803.1	2804.84	1.74	0.06%
Spinalcord	20.78	20.99	0.21	1.00%
Subglottic larynx	6.14	6.17	0.02	0.32%
Superior pharyngeal constrictors	15.65	16.53	0.88	5.32%
Supraglottic larynx	16.80	17.12	0.32	1.87%
Surg bed PTV 60 Gy	258.53	259.78	1.25	0.48%
Surgical bed CTV	119.95	118.59	1.36	1.15%
Thyroid	10.85	11.37	0.52	4.57%

**Table A2:** PTV overlap (cm^3^) calculation comparisons of DVH Analytics and Pinnacle^3^

ROI name	DVH Analytics	Pinnacle^3^	Abs. Diff.	Rel. Diff.
Base of tongue	4.49	4.53	0.04	0.88%
Brachial plexus total	11.90	12.07	0.17	1.41%
Brain	0.00	0.00	0.00	–
Brainstem	0.00	0.00	0.00	–
Cervico‐thoracic esophagus	0.00	0.00	0.00	–
Cochlea l	0.00	0.00	0.00	–
Cochlea r	0.00	0.00	0.00	–
Constrictors total	7.57	7.70	0.13	1.69%
Ext	573.83	574.36	0.53	0.09%
Eye l	0.00	0.00	0.00	–
Eye r	0.00	0.00	0.00	–
Glottic larynx	1.24	1.27	0.03	2.36%
Inferior pharyngeal constrictors	0.00	0.00	0.00	–
Larynx total	3.02	3.10	0.08	2.58%
Left brachial plexus	5.07	5.15	0.08	1.55%
Lips	0.00	0.00	0.00	–
Mandible	3.67	3.79	0.12	3.17%
Middle pharyngeal constrictors	0.10	0.11	0.01	9.09%
Opticchiasm	0.00	0.00	0.00	–
Opticnerve l	0.00	0.00	0.00	–
Opticnerve r	0.00	0.00	0.00	–
Oralcavity	0.17	0.19	0.02	10.53%
Parotid l	0.00	0.00	0.00	–
Parotid r	6.67	6.72	0.05	0.74%
Parotid total	6.67	6.72	0.05	0.74%
Pituitary	0.00	0.00	0.00	–
Posterior cricoid esophagus	0.00	0.00	0.00	–
Presurgery gtv	12.09	12.18	0.09	0.74%
PTV 54 Gy	244.45	244.66	0.21	0.09%
PTV 56 Gy	69.82	69.87	0.05	0.07%
Right brachial plexus	6.83	6.91	0.08	1.16%
rt. neck bst PTV 6 Gy	20.68	20.74	0.06	0.29%
Skin	476.16	476.64	0.48	0.10%
Spinalcord	0.00	0.00	0.00	–
Subglottic larynx	0.59	0.60	0.01	1.67%
Superior pharyngeal constrictors	7.48	7.58	0.10	1.32%
Supraglottic larynx	1.19	1.23	0.04	3.25%
Surg bed PTV 60 Gy	259.63	259.78	0.14	0.05%
Surgical bed CTV	118.46	118.59	0.13	0.11%
Thyroid	1.31	1.51	0.20	13.25%

**Table A3:** Minimum distance (cm) to PTV calculation/measurement comparisons of DVH Analytics and Pinnacle^3^

ROI name	DVH Analytics	Pinnacle^3^	Abs. Diff.	Rel. Diff.
Base of tongue	0.00	0.00	0.00	–
Brachial plexus total	0.00	0.00	0.00	–
Brain	0.90	0.87	0.03	3.45%
Brainstem	1.02	1.03	0.01	0.97%
Cervico‐thoracic esophagus	0.48	0.49	0.01	2.04%
Cochlea l	4.17	4.51	0.34	7.54%
Cochlea r	1.82	1.52	0.30	19.74%
Constrictors total	0.00	0.00	0.00	–
Eye l	5.51	5.45	0.06	1.10%
Eye r	4.42	4.37	0.05	1.14%
Glottic larynx	0.00	0.00	0.00	–
Inferior pharyngeal constrictors	0.00	0.00	0.00	–
Larynx total	0.00	0.00	0.00	–
Left brachial plexus	0.00	0.00	0.00	–
Lips	4.00	3.88	0.12	3.09%
Mandible	0.00	0.00	0.00	–
Middle pharyngeal constrictors	0.00	0.00	0.00	–
Opticchiasm	4.50	4.18	0.32	7.66%
Opticnerve l	4.70	4.64	0.06	1.29%
Opticnerve r	4.50	4.21	0.29	6.89%
Oralcavity	0.00	0.00	0.00	–
Parotid l	0.12	0.00	0.12	–
Parotid r	0.00	0.00	0.00	–
Parotid total	0.00	0.00	0.00	–
Pituitary	3.60	3.28	0.32	9.76%
Posterior cricoid esophagus	0.58	0.47	0.11	23.40%
Right brachial plexus	0.00	0.00	0.00	–
Spinalcord	1.09	1.09	0.00	0.00%
Subglottic larynx	0.00	0.00	0.00	–
Superior pharyngeal constrictors	0.00	0.00	0.00	–
Supraglottic larynx	0.00	0.00	0.00	–
Thyroid	0.00	0.00	0.00	–
